# Current Status of Forest Health Policy in the United States

**DOI:** 10.3390/insects10040106

**Published:** 2019-04-12

**Authors:** Kamal J. K. Gandhi, Faith Campbell, Jesse Abrams

**Affiliations:** 1 D.B. Warnell School of Forestry and Natural Resources, University of Georgia, 180 E Green Street, Athens, GA 30602, USA; Jesse.Abrams@uga.edu; 2 Center for Invasive Species Protection, 8208 Dabney Avenue, Springfield, VA 22152, USA; phytodoer@aol.com; 3 Savannah River Ecology Laboratory, P.O. Drawer E, Aiken, SC 29802, USA

**Keywords:** federal policy, forest disease, forest insects

## Abstract

Federal policies related to forestry and forest health (specifically, insects and diseases) have the potential to affect management practices, terms of international and interstate trade, and long-term sustainability and conservation. Our objectives were to review existing federal policies, the role of federal agencies in managing forest health, and guidance for future policy efforts. Since the 1940s, various federal policies relevant to forest health have been established, and several US Department of Agriculture (USDA) agencies have been empowered to assist with prevention, quarantine, detection, management, and control of insects and diseases. Overall, our review showed that relatively few national policies directly address forest health as a stand-alone objective, as most of them are embedded within forestry bills. Federal funding for forest health issues and the number of personnel dedicated to such issues have declined dramatically for some agencies. Concomitantly, native species continue to gain pestiferous status while non-native species continue to establish and cause impacts in the US. To enhance our ability and capacity to deal with current and future threats, concerted efforts are needed to advocate for both resources and stand-alone policy tools that take seriously the complexity of emerging sustainability challenges in both private and public forestlands.

## 1. Introduction

Natural and planted forests encompass ~300 million hectares and ~32% of total land area in the US. [1]. Of these forests, 56% are privately owned and the rest are publicly owned, that is, managed by local, tribal, state, and federal governments [1]. The western region contains the majority of the country’s public forestlands, managed for a mix of uses including timber production, recreation, and conservation, while the eastern US is dominated by private forests, some of which are heavily utilized for timber production [2]. This leads to vastly different management goals, objectives, and approaches especially under variable political conditions. However, a common goal for these various types of forests across the nation is to maintain their long-term productivity and resilience, as demands and pressure on resources grow with time.

America’s extensive and diverse forests, including those in urban areas, provide a basis for multiple uses, including but not limited to timber and fiber extraction; non-timber products; recreation; wildlife habitat; clean water, soil, and air; and carbon sequestration, and are of high economic values to local communities, worth billions of dollars each year [3,4,5,6]. Further, US forests are rich repositories of local biodiversity with thousands of endemic plant and animal species—many of which are critical to maintaining extant forest processes and patterns [7]. For example, fire-adapted pine species such as longleaf (*Pinus palustris* Mill.) and ponderosa (*Pinus ponderosa* Dougl. ex Laws.) pines have unique biotic components associated with them, and the Appalachian Mountains have high rates of endemism for salamander and bird species [6,7,8,9]. Long-term conservation, sustainability, and resilience of the US forests is, therefore, of high importance both economically and ecologically.

Historically, US forests have been subjected to both natural and anthropogenic disturbances [3,10]. Examples of natural disturbances include wildfires, flooding, landslides, ice storms, windstorms, and native insects and diseases [11,12]. Anthropogenic disturbances include logging, conversion to agriculture and/or urbanization, non-native insects/diseases, and forest clearing for industrial uses, which can result in fragmentation, isolation, and loss of original habitat [13]. Climate change interacts with these disturbances in complex ways, increasing stress on trees and associated forest species [14]. Forests tend to be adapted and resilient to natural disturbances of variable frequency, intensity, and severity, but their successional and recovery pathways may differ greatly when anthropogenic disturbances are more dominant and common on the landscape. Interactions between various natural and anthropogenic disturbances or “compounded” disturbances (e.g., salvage logging after a windstorm or wildfires) are currently determining landscape heterogeneity, and patterns and processes that are evident in the US forests [15,16].

**Figure 1:**
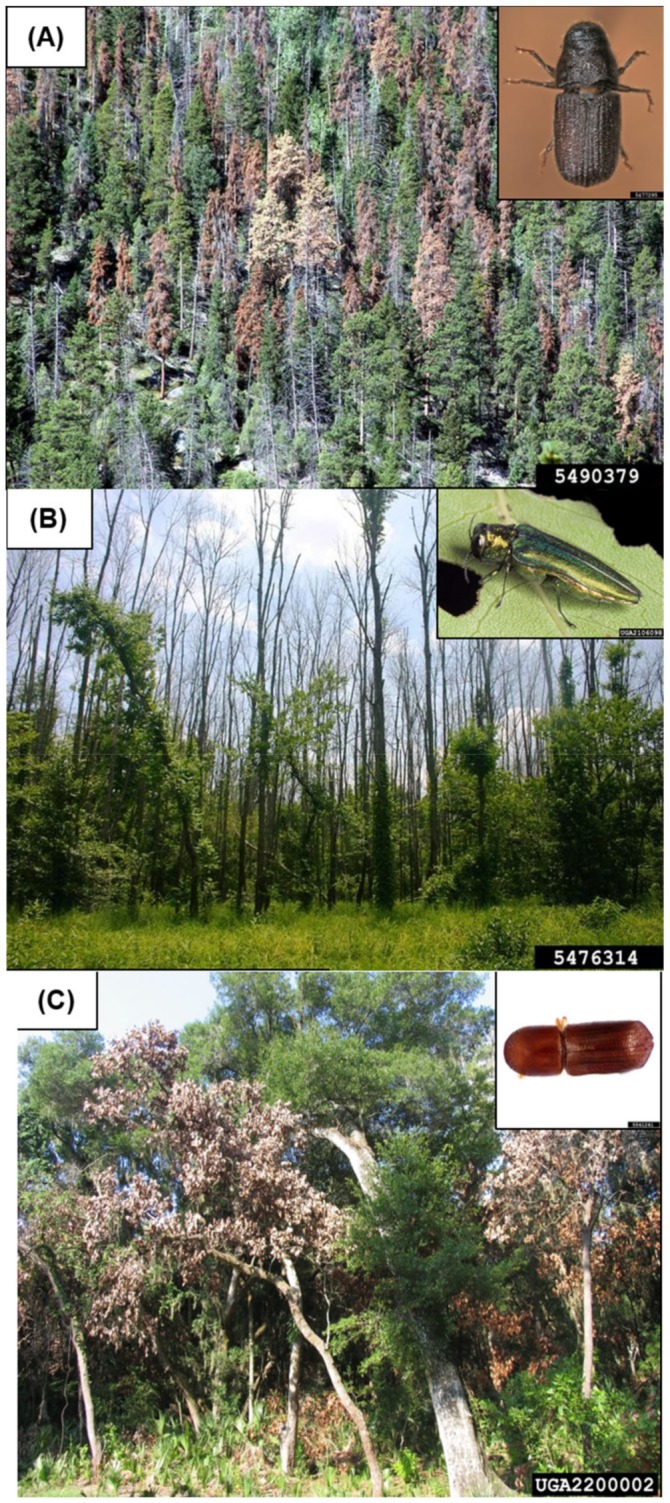
Examples of tree mortality caused by three native and non-native insects and diseases: (**A**) Mountain pine beetle (image credits: Whitney Cranshaw, Colorado State University, Bugwood.org and Javier E. Mercado, Bark Beetle Genera of the US, US Department of Agriculture (USDA), Animal and Plant Health Inspection Service (APHIS), Plant Protection and Quarantine (PPQ), Bugwood.org). (**B**) Emerald ash borer (image credits: Christopher Asaro, Virginia Department of Forestry, Bugwood.org and David Cappaert, Bugwood.org). (**C**) Redbay ambrosia beetle (image credits: Joseph Benzel, Screening Aids, USDA APHIS PPQ, Bugwood.org and Albert Mayfield, USDA Forest Service, Bugwood.org).

Insects and diseases, both native and non-native, have great potential to alter and affect tree and forest health with cascading ecological impacts (Figure 1). Native insect and disease species are natural disturbance agents to which ecosystems are adapted [17], whereas non-native species are a major cause of demise of major tree genera and loss of biodiversity from which little recovery is possible [18,19]. Prominent examples of non-native species that have devastated the forested landscapes and altered them forever include loss of elms (*Ulmus americana* L.) due to Dutch elm disease (*Ophiostoma novo-ulmi* Brasier vectored by *Scolytus multistriatus* Marsh.), whitebark pine (*Pinus albicaulis* Engelm.) due to blister rust (*Cronartium ribicola*. J.C. Fisch.), ash (*Fraxinus* spp.) due to emerald ash borer (*Agrilus planipennis* Fairmaire), hemlocks (*Tsuga* spp.) in eastern US due to hemlock woolly adelgid (*Adelges tsugae* Annand), and red bays (*Persea borbonia* (L.) Spreng.) due to laurel wilt disease (*Raffaelea* spp. vectored by *Xyleborus glabratus* Eichhoff) (Figure 1) [20,21,22,23,24,25]. In many landscapes, both native and non-native invasive species may cause impacts at the same time and by interacting with each other, thus, leading to larger and more managerially complex forest health problems.

Public policies are enacted to address an issue or a problem with implications for public values and interests [26,27]. Public policies intended to influence private behavior may include punitive (regulatory) measures, incentives (e.g., subsidies), learning and capacity-building tools, and symbolic tools [28]. A separate suite of public policies set objectives, provide funding, and authorize the use of particular tools for public agencies and the public resources (e.g., public lands) they are entrusted to manage [27,29]. These policies are enacted, implemented, and interpreted by the legislative, executive, and judicial government branches, providing direction and resources for public and private behavior. Through effects on the global flow of goods, the management of forests and forest-associated species, and the generation and diffusion of knowledge, public policies can greatly influence the nature and severity of insect and disease impacts on forests. With the current increased interest in policy issues related to forest health (especially under shrinking funding for control, management, and research activities), our objectives are to: (1) provide a historical context and an overview of the roles of major federal agencies in managing insects and diseases; (2) outline major policies related to forest health and their possible impacts; and (3) identify gaps where new policies can play a major role in enhancing forest health in the US. We are particularly interested in laws on forest management practices, those that influence activities of the government agencies, and those that regulate the movement of insects and diseases within and outside the country [30].

## 2. Background and Historical Context 

Forest managers may treat native and non-native insects and diseases alike, viewing both as equally unwelcome invaders whose presence should be minimized or eliminated. Contemporary understanding of forest dynamics, however, suggests that native insects and diseases can play important roles in stand dynamics, nutrient cycling, and the creation of stand- and landscape-level heterogeneity and that some control measures may have reduced overall forest resilience [31,32]. At the same time, the unprecedented scale and impacts of recent insect outbreaks such as the mountain pine beetle (*Dendroctonus ponderosae* Hopkins) in the Rocky Mountains, the American Southwest, and the Sierra Nevada, and southern pine beetle (*Dendroctonus frontalis* Zimmermann) in the northeastern states point to the importance of both climate change and forest conditions in driving insect activity outside of historic patterns of variability [17,25,32,33]. Such impacts from various native bark beetles are estimated to be 5.2 million ha of tree mortality in western forests between 1997 and 2012, and these eruptions continue to occur each year [34].

The continued establishment and spread of non-native forest insects create additional impacts and damage to those created by native insects. For example, [35] estimated that the annual cost of wood and phloem feeding non-native insects alone includes at least $1.7 billion in government expenditures and $830 million in household expenditures and residential property value loss. These economic values are an underestimation since loss of ecosystem services such as cascading impacts on native biodiversity and changes in stand structure and attributes, soil, air, and water conditions are rarely quantified. A greater emphasis is being placed on ecological impacts in recent years for restoration purposes; for example, many more studies exist on ash tree dieback since the 2000s (summarized by [36]) than on the loss of elms and chestnuts (*Castanea dentata* (Marsh.) Borkh) since the mid-20th century.

As our collective understanding of forest insect dynamics advances, policy tools and mandates informed by high-quality scientific information are essential for sustainable forest management [37]. Public policy has a central role to play in managing forest health, for several reasons. The first is that the cross-boundary nature of insect movement and impacts presents a collective action problem to which individual landowners are unlikely to respond effectively in isolation [38]. Public policy creates a coordinating mechanism for research, monitoring, and action on insects and their impacts. Second, the federal government is the nation’s largest forest owner via the National Forest System, Bureau of Land Management lands, and other land management agencies. Some of the largest and most destructive insect outbreaks in recent history have occurred in western mountain regions dominated by federal ownership [17]. Third, public policies govern the detection, prevention, and management of non-native forest pests and pathogens that enter the country through the pathway of importation of goods. Finally, the federal government has had a longstanding leadership role in research, monitoring, and treatment for both native and non-native insect and disease species across ownership boundaries, as detailed in the following sections.

## 3. Role of the US Department of Agriculture (USDA) Forest Service

The mission of the USDA Forest Service is “to sustain the health, diversity, and productivity of the Nation’s forests and grasslands to meet the needs of present and future generations” [39]. Linked to this mission is the wise stewardship of forests through conservation, protection, and management using the concept of multiple use. The USDA Forest Service also strives to provide technical and financial assistance to landowners and foresters (both nationally and internationally), and develops technical and scientific knowledge to further enhance its mission. With such diverse objectives, the USDA Forest Service has had a major impact on our nation’s forests at many levels.

Federal forest insect policy reflects a longstanding model of public–private cooperation in which the federal government provides technical and financial resources for treating both native and non-native forest insects and diseases across ownership boundaries. Federal agencies and federally funded scientific research have been central components of managing forest insects and diseases in the US. For the early US Department of Agriculture (under which the USDA Forest Service is located), eliminating bark beetles, defoliators, and other agents of tree mortality was a logical component of a larger vision of a regulated forest efficiently producing timber for human needs [40]. The federal government was sending scientists from the Bureau of Entomology on investigative missions as early as 1899 when Andrew Delmar Hopkins investigated insect concerns on forests in the Pacific Northwest [40]. The young Forest Service was experimenting with new insect detection and control strategies on federal lands from the early years of the 20th century [41]. The federal government consolidated its authority to conduct cross-boundary research and management of forest insects in 1947 via the Forest Pest Act [42]. This authority was later affirmed and expanded via policies such as:

- The Forest and Rangeland Renewable Resources Research Act of 1978—which builds on a similar act in 1974 [43]. This act states that the federal government has a substantial role in the “health, productivity, and sustainability of the forests and rangeland of the United States” including on public and private lands. Further, the international component of forestry was acknowledged, and research activities were expanded to a global scale. The principal revisions relating to forest health are found in Section 3(a)(3) (16 United States Code (U.S.C.) 1642), which authorizes research for protecting renewable resources from “fires, insects, diseases, noxious plants, animals…” [44]. This act also required the USDA to conduct an inventory of forest resources. For example, the Forest Inventory Analysis (FIA), which in addition to reporting on the current status and trends of forests (species, size, type, growth, harvest, etc.), also includes an inventory of damage caused by insects and diseases [45].
- The Healthy Forests Restoration Act (HFRA) of 2003 (House of Representatives (H.R.) 1904), (16 U.S.C. 6501–6502, 6511–6518, 6541–6542, 6571–6578)—aimed primarily at addressing increases in the scale and impact of wildfires and insect outbreaks in forests nationwide [46]. Specifically, Title I Hazardous Fuels Reduction on Federal Lands established new environmental planning and analysis procedures for hazardous fuel reduction projects including those caused by insect epidemics on at-risk National Forest System and Bureau of Land Management lands, and provided other authorities and direction to help reduce hazardous fuel and restore forests and rangelands on lands of all ownerships. Title IV, Insect Infestations and Related Diseases, promoted the collection of monitoring data on insects and diseases that cause large-scale damage through partnerships with state, university, and private entities to assist with maintaining forest health. Section 404 allowed for “applied silvicultural assessments”, expedited treatments of areas of up to 1000 acres of federal land considered to be infested or at high risk of infestation.
  This act was amended in the 2014 Farm Bill; Title VI, Section 602 (Designation of Treatment Areas) established priorities for projects in areas that would reduce and ameliorate insect and disease outbreaks [47]. Similar to areas designated for applied silvicultural assessments, these treatment areas were categorically exempt from determination of environmental impact significance under the National Environmental Policy Act (NEPA). Up to $200 million was authorized annually to carry out such treatments in National Forests. The 2014 Farm Bill also included a provision that amended HFRA to require the Secretary of Agriculture to designate landscape-scale insect and disease treatment areas in response to a petition by the relevant state governor; an additional categorical exclusion under NEPA was included in the bill [47].
- The “Wyden Amendment” (Public Law (P.L.) 105–277, Section 323) authorizes the USDA Forest Service to enter into cooperative agreements with “federal, tribal, state and local governments, private and nonprofit entities and landowners” to benefit federal lands and related investments at the watershed scale [48]. These agreements may support or conduct invasive species management activities on aquatic and terrestrial areas owned by non-USDA Forest Service entities to benefit and protect national forestlands and other resources within a watershed at risk from invasive species.

Federal policy content specifically focused on forest insects has become more common in recent proposed and enacted congressional legislation [32,49], largely driven by the growing scale and incidence of outbreaks nationwide. In many ways, questions of insect policy have become embroiled in larger debates regarding the purposes of federal forestlands and the prudence of expediting silvicultural treatments as a response to both insect and wildfire risk [32,49]. Indeed, the concept of “forest health” has itself emerged as a highly contested concept in policy debates over federal forest policy [17,29]. As recently as December 2018, Executive Order 13855 specifically identified insects as a contributing factor to the loss of forest and rangeland health nationwide and ordered the Secretaries of Interior and Agriculture to “identify salvage and log recovery options from lands damaged by fire during the 2017 and 2018 fire seasons, insects, or disease” [50].

In addition to formal federal policymaking responses to forest insect outbreaks, the creation of regional place-based networks as a means of providing direction and resources for outbreak response has been observed in some geographies [51,52,53]. These networks function to build consensus regarding management response, attract public investments in response plans, innovate practical solutions to problems encountered in implementation, and leverage the capacity existent among the various federal, state, local governmental, private, and civil society network partners. The limited research to date on these networks indicates that they may take advantage of recent forest policy tools and authorities to leverage greater treatment outcomes once consensus has been established [51,53].

Overall, the task and mandate of the USDA Forest Service to protect and maintain forests (especially in National Forests) from forest health issues as defined by federal policy has increased, while the funding and resources to accomplish it have significantly decreased. This trend has been in place for at least two decades, and greatly hampers the recovery and restoration of forest stands. In the case of native insects achieving pest status, some of the forest health issues appear to be emerging primarily from the National Forests in the southeastern region [54]. An example is that of the Oconee Ranger District in the Chattahoochee National Forest System in Georgia, which has had extensive *Ips* and southern pine beetle outbreaks, while private properties surrounding the District had low levels of bark beetle infestations (personal observations). Similar trends have been observed for southern pine beetle outbreaks in some National Forests in Mississippi [54].

Through its Forest Health Management (FHM) program under the State and Private Forestry program of Forest Health Protection (FHP), the USDA Forest Service assists non-federal government agencies—state and municipal—in evaluating and providing early warning on threats from both native and non-native forest pests [55]. The FHM program provides data, reports, maps, and consultation with experts to natural resource managers, landowners, policymakers, researchers, and analysts. For example, the $73 million Southern Pine Beetle Prevention Initiative, which (among other outcomes) led to landowner incentive payments to conduct pine plantation thinning and prescribed burning to reduce susceptibility to the southern pine beetle [56]. However, funding for the USDA Forest Service—FHM program has been reduced significantly in recent years. In Fiscal Year 2011, Forest Health Protection received $132 million. The appropriation fell to $99.6 million by FY2016, then to $93.8 million in FY2018 [57,58]. Thus, in the face of rising numbers of pests that need to be addressed, funding levels have fallen substantially.

The USDA Forest Service FHP also provides funding for various forest health projects related to evaluating the risk of insects and diseases, pesticide impact assessment, biocontrol of invasive plants, and new technology on the prediction and management of insects and diseases [59]. Many of these grants serve as a backbone for forest health projects developed collaboratively between universities and federal and state entities. Similar to other programs, funding has been drastically cut in the last decade, leading many scientists to search for alternative funding to achieve the same goals.

**Figure 2:**
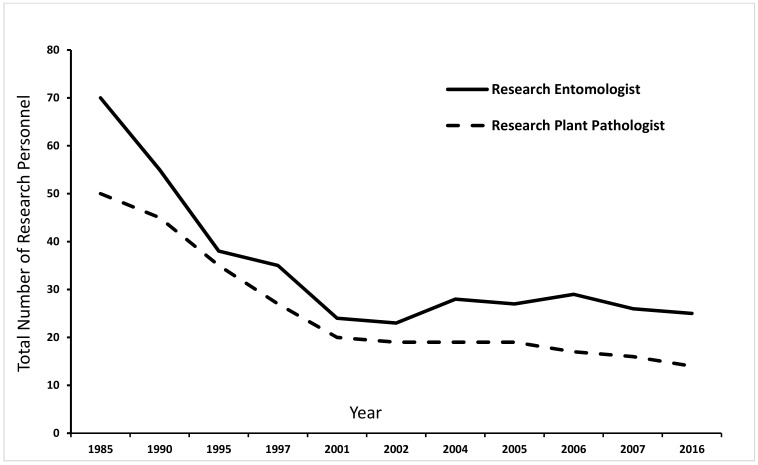
Total number of research entomologists and pathologists employed during 1985-2016 within the USDA Forest Service, Southern Research Station system. Credit: J. Guldin, USDA Forest Service (personal communication).

The USDA Forest Service Research and Development program funds in-house and extramural research on issues affecting the detection and management of insect pests. Funding for invasive species represents a small proportion of the total research and development budget and there is no specific line item for this function. Funding for research—both overall funding and funding targeting invasive species—has declined precipitously. Funding for invasive tree pests declined from $8 million in 2010 to $3 million in 2018. The Southern Research Station’s research division had 70 entomologists and 50 pathologists in the mid-1980s (Figure 2). By 2007, the numbers had declined to about 25 entomologists and 14 pathologists, an average of ~70% decline in number of dedicated research personnel which has had substantial impact on research activities (Figure 2). Similar trends are also present in other research stations across the country.

## 4. Roles of the USDA Animal and Plant Health Inspection Service (APHIS) and Agriculture Research Services (ARS)

The US Department of Agriculture’s Animal and Plant Health Inspection Service (USDA APHIS) has the principal responsibility for managing non-native insects and pathogens that attack native trees [60]. This includes preventing pests’ initial introduction, implementing quarantines and other programs intended to eradicate or at least prevent further spread of pests that are introduced, and management of pests that have established.

USDA APHIS’ programs are carried out primarily under the authority of the Plant Protection Act (PPA) of 2000 (7 U.S.C. §7701, et seq. (2000)) [61]. This statute authorizes the agency to regulate any living stage of any of the following that can directly or indirectly injure, cause damage to, or cause disease to any plant or plant product such as protozoans, nonhuman animals, parasitic plants, bacterium, fungus, virus, viroid, and infectious agents [61]. Also regulated under the PPA are biocontrol agents, organisms altered by certain biotechnical (genetic engineering) procedures, and noxious weeds.

The statute provides the legal foundation for USDA APHIS to regulate the importation, exportation, and interstate movement of plant pests and articles (including plants) that could transport these pests [62]. Regulation of pests within individual states is carried out by that state except in cases when the Secretary of Agriculture declares an extraordinary emergency. The Plant Protection Act prohibits importation or movement in interstate commerce (including by mail) of any plant pest unless otherwise authorized under a permit issued by USDA APHIS. Policies and regulations must be based on sound science, transparent, and accessible. USDA APHIS, thus, has broad authority to take a range of actions including holding, seizing, putting under quarantine, applying various remedial measures to, destroying, or otherwise disposing of any plant, plant product, other article, or means of conveyance, that threatens to move a regulated plant pest. USDA APHIS may also order the owner of any of the regulated articles (or their agents) to take required actions.

USDA APHIS inspects imports of living plants at one of its 16 Plant Inspection Stations [63]. All other imports such as fruits, vegetables, and grains for consumption and miscellaneous merchandise in packaging made from wood are inspected by the US Bureau of Customs and Border Protection (CBP), a division of the Department of Homeland Security [64]. In carrying out these inspections and associated treatment or disposal decisions, the CBP follows protocols and rules established by the USDA APHIS, thus broadening their authority.

Similar to other federal agencies, USDA APHIS’ actual efforts to manage plant pests—especially those that threaten tree species—have been severely hampered by funding shortfalls. Funding for the management of “tree and wood pests” has declined from $85 million in 2009 to $54 million in 2018. At the same time, the number of pests has risen and the extent of known infestations and difficulty of addressing them have also increased. Among high-risk insect pests that USDA APHIS has not officially designated as “quarantine pests” are redbay ambrosia beetle, spotted lanternfly (*Lycorma delicatula* (White)), and polyphagous and Kuroshio shot hole borers (*Euwallacea* spp.). USDA APHIS does provide some funding to address these pests, especially through grants funded through the Plant Pest and Disease Management and Disaster Program (§7721 of the Plant Protection Act).

Under 7 U.S.C. §7772, USDA APHIS also has authority to access “emergency” funds from funds available to the Department of Agriculture. These funding sources are not subject to annual appropriations. However, the agency’s access is tightly controlled by the Office of Management and Budget. At the beginning of 2018, e.g., USDA APHIS accessed $17 million in emergency funding to address the expanding spotted lanternfly outbreak [65].

The USDA Agriculture Research Services or USDA ARS aims to find and transfer solutions to major agricultural issues in the US. Their mandate is much broader than the USDA Forest Service as they mostly focus on nutritional and food crop systems but do include “natural resource base and the environment” as part of their mission statement [66]. Under their National Programs 303—Plant Diseases and 304—Crop Protection and Quarantine, the USDA ARS scientists work on new pests and pathogens that are detected within the country to provide technical information related to their detection, monitoring, epidemiology, and management [67].

## 5. Farm Bill

The 2008 Farm Bill (P.L. 110–234) established the Plant Pest and Disease Management and Disaster Program, which provided permanent funding drawn from the Commodity Credit Corporation to support enhanced pest analysis, improved pest surveillance, improved pest identification and technology enhancement, targeted domestic surveillance, enhanced mitigation capabilities, safeguard nursery production, and outreach [68]. The Agricultural Act of 2014 (known as the 2014 Farm Bill, P.L. 113–170) amended the Plant Protection Act to combine the National Clean Plant Network and the Plant Pest and Disease Management and Disaster Program, making them permanent [69]. It raised the authorized funding level for the combined programs to $62.5 million per year from FY2014–FY2017 and $75 million per year in FY2018 and beyond. The program now operates under Section 7721 of the Plant Protection Act (7 U.S.C. Section 7721). During 2014–2017, ~$63 million in Commodity Credit Corporation funding was provided under this program [70]. In 2019 alone, the Plant Pest and Disease Management and Disaster Program funded about $60 million in programs related to enhancing plant pest/disease analyses and surveys, targeting domestic inspection activities at vulnerable points, pest identification and detection technology, enhancing mitigation capabilities, and rapid response [71]. Hence, the Farm Bill provides a substantial investment in the detection, quarantine, and control of pests and diseases in the US each year with variable funding levels. Over the past decade (as assessed from project reports), only about 10% of these funds has supported projects focused on forest pests and pathogens, and there is need for more funding targeted at critical forest health needs.

## 6. Conclusions

During the last few decades, there have been some major policies embedded as part of the larger farm and forestry bills that have indirectly and directly affected forest health issues in the US. While it is hard to quantify how these polices have directly shaped forest health and its management, such investments have been critical to allowing detection, control, and management to proceed as needed at multiple levels (e.g., [72]) in addition to providing solution-driven research resources through collaborations with diverse agencies. It is also clear that these few policies are not enough—and much more are needed especially under variable climatic conditions to empower scientists and managers at all levels. Even in the wake of devastating pest outbreaks such those by bark beetles on federal lands, the federal policy response was quite weak [51]. Many forest policies, in fact, appear to be static, and changes are made largely to budgetary allocations [26]. In contrast, many more bills are proposed, very few of them move forward to become policy, and even fewer result in funding appropriations. For example, H.R. 4976—Empowering State Forestry to Improve Forest Health Act of 2018—was proposed with an objective to “improve forest health and forest ecosystems, including addressing native, nonnative, and invasive pests”; this bill did not pass [73]. From identifying an issue, working with Congress to propose a bill, to policy establishment and implementation is akin to turning around a massive ship, and this is certainly true for the forest health field.

Insect and disease policy on federal lands has increasingly reflected prevailing social and political contestations regarding the proper role of human intervention in relatively natural forest systems. The emergence of regional governance networks with access to various tools for planning, responding to, and recovering from insect outbreaks—as well as to scientific information—suggests possible constructive pathways forward through conflict to achieving adaptive responses. However, long-term declines in funding for federal forest management (especially when major portions of the USDA Forest Service funding are spent fighting fires each year) [58] complicate the ability of such networks to implement adaptive management at appropriate scales without substantial investments from other sources. USDA National Institute of Food and Agriculture, Agriculture and Food Research Initiative (USDA AFRI), another major funding agency that supports applied research, does not have a stand-alone program on forest health, which dilutes the funding pool through competition with other fields. Similarly, there are several commodity-driven funding programs focused on crop and orchard plants, but none on forest trees, which are also critical commodity products in many regions in the US (particularly the southeastern region).

Introduction, establishment, and spread of non-native insects and diseases are managed by a separate USDA agency, the APHIS. Under the Plant Protection Act, USDA APHIS operates under mandates to both facilitate international trade and “reduce, to the extent practicable” the associated risk of pest introductions (PPA Sec. 7701 (3))—this creates a balancing situation. Clearly, greater and more comprehensive phytosanitary measures are needed as many sources demonstrate that forest pest and pathogen arrivals and introductions continue to occur in the US [74,75,76,77,78]. Management of pests in these situations is also impeded by several factors including the difficulty in detecting them before they are established over a significant area, poor understanding of the insect’s life cycle and spread potential, and sometimes opposition to management strategies such as removing of live trees and aerial spraying of pesticides or pheromone-infused particles [77].

Reference [37] reviewed the impacts of non-native insects and diseases in the US and provided policy suggestions that may reduce their arrival and establishment by focusing on their major pathways and imported material (e.g., wood packaging and live-plant trade). Examples of suggestions include strengthening overseas relationships and enforcement of regulatory actions (home and abroad), increasing funding for all aspects of the invasion process (e.g., early detection, quarantine, eradication, control, etc.), and improved data collection and dissemination with input from an advisory board of scientists. Similar policy recommendations for native pestiferous species are notably scarce or absent. The combination of anthropogenic influences on forest conditions and on climatic patterns has allowed some native insects and diseases to behave—and to affect forests—in novel and unexpected ways through range expansion. Adaptive approaches to insect management will become more important than ever, and public policies will be important in setting the stage for forest restoration activities. Without the strong federal support, our forests will continue to be devasted, with major implications for their long-term resilience.

Many scientists have opportunities to be involved in policy advocacy, depending upon the guidelines of their respective agencies and institutions [79,80]. Advocacy can include one-on-one contact (visit and/or call) with local legislatures, visiting them in Washington DC, and working through their stakeholders to reach lawmakers. Further, many scientific societies such as the Entomological Society of America (ESA) and the American Association for the Advancement of Science (AAAS) have well-developed and successful science policy fellowships. These programs acknowledge that scientists can play important roles in directing new laws and policies in their field, and that their expertise is needed for effective decision-making [81,82]. These training programs also provide insights into the workings of the government, and federal funds being allocated to various fields, thus allowing for greater visibility of the available funding pool. Grassroots initiatives on advocating forest health issues are also being developed, for example for providing greater funding to insect and disease–host resistance research by several groups [83]. We expect that some of these local efforts will convene while maintaining their individual missions and agendas, for greater positive impact on forest health funding in the future.

Overall, we recommend a push for stand-alone major policy and funding efforts focused solely on enhancing the health of our forests. Such efforts will rebuild our capacity to respond effectively to insect and disease outbreaks, as they happen in real time. Policies that strengthen the research, outreach, and managerial capacity of federal agencies while also providing support to regional multi-stakeholder networks of practice would go a long way toward improving the acceptability and effectiveness of insect and disease management strategies and their impacts on US forests.

## References

[1] USDA Forest Service US Forest Facts and Historical Trends. USDA Forest Service, FIA Publication. https://www.fia.fs.fed.us/library/brochures/docs/2000/ForestFactsMetric.pdf.

[2] USDA Forest Service Who Owns America’s Forests?. https://www.nrs.fs.fed.us/pubs/inf/NRS-INF-06-08.pdf.

[3] Wear D.N., Gries J.G. (2002). Southern Forest Resource Assessment—Summary Report.

[4] Moore R., Williams T., Rodriguez E., Hepinstall-Cymmerman J. (2013). Using non-market valuation to target conservation payments: An example involving Georgia’s private forests. J. For..

[5] Binder S., Haught R.G., Polasky S., Warziniak T., Mockrin M.H., Deal R.L., Arthaud G. (2017). Assessment and Valuation of Forest Ecosystem Services: State of the Science Review.

[6] Chamberlain J.L., Bush R.J., Hammett A.L., Araman P.A. (2002). Eastern National Forests: Managing for nontimber products. J. For..

[7] Jenkins C.N., Van Houtan K.S., Pimm S.L., Sexton J.O. (2015). US protected lands mismatch biodiversity priorities. Proc. Natl. Acad. Sci. USA.

[8] Trani M.K. (2002). Terrestrial Ecosystem. Southern Forest Resource Assessment.

[9] Noss R.F., Platt W.J., Sorrie B.A., Weakley A.S., Means D.B., Costanza J., Peet R.K. (2015). How global biodiversity hotspots may go unrecognized: Lessons from the North American Coastal Plain. Div. Dist..

[10] Johnstone J.F., Allen C.D., Franklin J.F., Frelich L.E., Harvey B.J., Higuera P.E., Mack M.C., Meentemeyer R.K., Metz M.R., Perry G.L. (2016). Changing disturbance regimes, ecological memory, and forest resilience. Fron. Ecol. Environ..

[11] Gandhi K.J.K., Gilmore D.W., Katovich S.A., Mattson W.J., Spence J.R., Seybold S.J. (2007). Physical effects of weather disturbances on the abundance and diversity of forest insects in North American forests. Environ. Rev..

[12] Flower C.E., Gonzalez-Meler M.A. (2015). Responses of temperate forest productivity to insect and pathogen disturbances. Ann. Rev. Plant Biol..

[13] Foster D.R., Aber J.D., Melillo J.M., Bowden R.D., Bazzaz F.A. (1997). Forest response to disturbance and anthropogenic stress. BioScience.

[14] Trumbore S., Brando P., Hartmann H. (2015). Forest health and global change. Science.

[15] Gandhi K.J.K., Gilmore D.W., Katovich S.A., Mattson W.J., Zasada J.C., Seybold S.J. (2008). Catastrophic windstorm and fuel-reduction treatments alter ground beetle (Coleoptera: Carabidae) assemblages in a North American sub-boreal forest. For. Ecol. Manag..

[16] Seidl R., Thom D., Kautz M., Martin-Benito D., Peltoniemi M., Vacchiano G., Wild J., Ascoli D., Petr M., Honkaniemi J. (2017). Forest disturbances under climate change. Nat. Clim. Chang..

[17] Raffa K.F., Aukema B.H., Bentz B.J., Carroll A.L., Hicke J.A., Turner M.G., Romme W.H. (2008). Cross-scale drivers of natural disturbances prone to anthropogenic amplification: The dynamics of bark beetle eruptions. BioScience.

[18] Lovett G.M., Canham C.D., Arthur M.A., Weathers K.C., Fitzhugh R.D. (2006). Forest ecosystem responses to exotic pests and pathogens in eastern North America. BioScience.

[19] Gandhi K.J.K., Herms D.A. (2010). Direct and indirect effects of invasive exotic insect herbivores on ecological processes and interactions in forests of eastern North America. Biol. Invas..

[20] Herms D.A., McCullough D.G. (2014). Emerald ash borer invasion of North America: History, biology, ecology, impacts, and management. Ann. Rev. Entomol..

[21] Rentch J., Fajvan M.A., Evans R.A., Onken B. (2009). Using dendrochronology to model hemlock woolly adelgid effects on eastern hemlock growth and vulnerability. Biol. Invas..

[22] Tomback D.F., Achuff P. (2010). Blister rust and western forest biodiversity: Ecology, values and outlook for white pines. For. Path..

[23] Fraedrich S.W., Harrington T.C., Rabaglia R.J., Ulyshen M.D., Mayfield III A.E., Hanula J.L., Eickwort J.M., Miller D.R. (2008). A fungal symbiont of the redbay ambrosia beetle causes a lethal wilt in redbay and other Lauraceae in the southeastern United States. Plant Dis..

[24] Karnosky D.F. (1979). Dutch elm disease: A review of the history, environmental implications, control, and research needs. Environ. Conser..

[25] Lesk C., Coffel E., D’Amato A.W., Dodds K., Horton R. (2017). Threats to North American forests from southern pine beetle with warming winters. Nat. Clim. Chang..

[26] Cubbage F.W., Newman D.H. (2006). Forest policy reformed: A United States perspective. For. Pol. Econ..

[27] Cubbage F., Harou P., Sills E. (2007). Policy instruments to enhance multi-functional forest management. For. Pol. Econ..

[28] Schneider A., Ingram H. (1990). Behavioral assumptions of policy tools. J. Pol..

[29] Vaughn J., Cortner H., George W. (2005). Bush’s Healthy Forests: Reframing the Environmental Debate.

[30] Coulson R.N., Stephen F.M., Paine T.D. (2008). Impacts of Insects in Forest Landscapes: Implications for Forest Health Management. Invasive Forest Insects, Introduced Forest Trees, and Altered Ecosystems.

[31] Baskerville G.L., Gunderson L.H., Holling C.S., Light S.S. (1995). The Forestry Problem: Adaptive Lurches of Renewal. Barriers and Bridges to the Renewal of Ecosystems and Institutions.

[32] Six D.L., Biber E., Long E. (2014). Management for mountain pine beetle outbreak suppression: Does relevant science support current policy?. Forests.

[33] Keane R.E., Ryan K.C., Veblen T.T., Allen C.D., Logan J.A., Hawkes B. (2002). The Cascading Effects of Fire Exclusion in Rocky Mountain Ecosystems: A Literature Review.

[34] Hicke J.A., Meddens A.J., Kolden C.A. (2015). Recent tree mortality in the western United States from bark beetles and forest fires. For. Sci..

[35] Aukema J.E., Leung B., Kovacs K., Chivers C., Britton K.O., Englin J., Frankel S.J., Haight R.G., Holmes T.P., Liebhold A.M. (2011). Economic impacts of non-native forest insects in the continental United States. PLoS ONE.

[36] Klooster W.S., Gandhi K.J.K., Long L., Perry K.I., Rice K., Herms D.A. (2018). Ecological impacts of emerald ash borer in forests at the epicenter of the invasion in North America. Forests.

[37] Lovett G.M., Weiss M., Liebhold A.M., Holmes T.P., Leung B., Lambert K.F., Orwig D.A., Campbell F.T., Rosenthal J., McCullough D.G. (2016). Nonnative forest insects and pathogens in the United States: Impacts and policy options. Ecol. Appl..

[38] Schelhas J., Molnar J., Martín-García J., Diez Casero J.J. (2012). A Common-Pool Resource Approach to Forest Health: The Case of the Southern Pine Beetle. Sustainable Forest Management-Current Research.

[39] USDA Forest Service (2019). What We Believe. https://www.fs.fed.us/about-agency/what-we-believe.

[40] Furniss M.M. (2007). A History of Forest Entomology in the Intermountain and Rocky Mountain Areas, 1901 to 1982.

[41] Graham R.T., Asherin L.A., Battaglia M.A., Jain T.B., Mata S.A. (2016). Mountain Pine Beetle: A Century of Knowledge, Control Attempts, and Impacts Central to the Black Hills.

[42] Steen H.K. (2004). The U.S. Forest Service: A History.

[43] U.S. Senate Committee on Agriculture and Forestry (1974). S.2296—An Act to Provide for the Forest Service, Department of Agriculture, to Protect, Develop, and Enhance the Productivity and Other Values of Certain of the Nation’s Lands and Resources, and for Other Purposes. https://www.congress.gov/bill/93rd-congress/senate-bill/2296.

[44] U.S. Senate Committee on Agriculture, Nutrition and Forestry Forest and Rangeland Renewable Resources Research Act of 1978. https://www.agriculture.senate.gov/imo/media/doc/Forest%20And%20Rangeland%20Renewable%20Resources%20Research%20Act%20Of%201978.pdf.

[45] USDA Forest Service Resources Planning Act. https://www.nrs.fs.fed.us/fia/topics/rpa/.

[46] U.S. Senate and House of Representatives Healthy Forests Restoration Act of 2003. https://www.fs.fed.us/emc/applit/includes/hfr2003.pdf..

[47] USDA Forest Service Farm Bill Amendments. https://www.fs.usda.gov/detail/r1/forest-grasslandhealth/insects-diseases/?cid=stelprd3854365.

[48] U.S. Congress Public Law 105-277-Oct. 21, 1998. https://www.congress.gov/105/plaws/publ277/PLAW-105publ277.pdf.

[49] Abrams J., Huber-Stearns H., Palmerin M.L., Bone C., Nelson M.F., Bixler R.P., Moseley C. (2018). Does policy respond to environmental change events? An analysis of mountain pine beetle outbreaks in the western United States. Environ. Sci. Pol..

[50] White House. Executive Office of the President Promoting Active Management of America’s Forests, Rangelands, and Other Federal Lands to Improve Conditions and Reduce Wildfire Risk. https://www.whitehouse.gov/presidential-actions/eo-promoting-active-management-americas-forests-rangelands-federal-lands-improve-conditions-reduce-wildfire-risk/.

[51] Abrams J.B., Huber-Stearns H.R., Bone C., Grummon C.A., Moseley C. (2017). Adaptation to a landscape-scale mountain pine beetle epidemic in the era of networked governance: The enduring importance of bureaucratic institutions. Ecol. Soc..

[52] Petersen B., Wellstead A.M. (2014). Responding to forest catastrophe in the face of unprecedented forest challenges: the emergence of new governance arrangements. ISRN Econ..

[53] Bobzien C., Van Alstyne K. (2014). Silviculture across large landscapes: back to the future. J. For..

[54] Asaro C., Nowak J.T., Elledge A. (2017). Why have southern pine beetle outbreaks declined in the southeastern US with the expansion of intensive pine silviculture? A brief review of hypotheses. For. Ecol. Man..

[55] USDA Forest Service Forest Health Monitoring. https://www.fs.fed.us/foresthealth/protecting-forest/forest-health-monitoring/index.shtml.

[56] Nowak J., Asaro C., Klepzig K., Billings R. (2008). The southern pine beetle prevention initiative: Working for healthier forests. J. For..

[57] USDA Forest Service Fiscal Year 2018 Budget Overview. https://www.fs.fed.us/sites/default/files/usfs-fy18-budget-overview.pdf.

[58] USDA Forest Service FY 2019 Budget Justification. https://www.fs.fed.us/sites/default/files/usfs-fy19-budget-justification.pdf..

[59] USDA Forest Service Forest Health Protection. https://www.fs.fed.us/foresthealth/grants.shtml.

[60] USDA APHIS About APHIS. https://www.aphis.usda.gov/aphis/banner/aboutaphis.

[61] U.S. Congress Title IV—Plant Protection. https://www.aphis.usda.gov/brs/pdf/PlantProtAct2000.pdf.

[62] USDA APHIS Strategic Plan FY 2019–2023. https://www.aphis.usda.gov/about_aphis/downloads/aphis-strategic-plan.pdf.

[63] USDA APHIS Plant Inspections Stations: Protecting U.S. Agriculture from Pests and Diseases. https://www.aphis.usda.gov/publications/plant_health/bro-inspection-stations-printer-eng.pdf.

[64] USDA APHIS Import Export. https://www.aphis.usda.gov/aphis/ourfocus/importexport.

[65] USDA APHIS (2018). Perdue Announces Emergency Funding for Spotted Lanternfly in Pennsylvania. https://www.usda.gov/media/press-releases/2018/02/07/perdue-announces-emergency-funding-spotted-lanternfly-pennsylvania.

[66] USDA ARS (2018). About ARS. https://www.ars.usda.gov/about-ars/.

[67] USDA ARS The 2017 Annual Report on Science. https://www.ars.usda.gov/ARSUserFiles/00000000/NPS/OAA/2017%20ARS%20Annual%20Report%20on%20Science.pdf.

[68] U.S. Senate Committee on Agriculture, Nutrition and Forestry 2008 Farm Bill. https://www.agriculture.senate.gov/imo/media/doc/110-246%20-%20Food,%20Conservation,%20And%20Energy%20Act%20Of%202008.pdf.

[69] U.S. Congress The Agricultural Act of 2014. https://www.congress.gov/113/plaws/publ79/PLAW-113publ79.pdf.

[70] USDA APHIS (2018). Farm Bill Section 10007 Program, Frequently Asked Questions (FAQs). https://www.aphis.usda.gov/plant_health/farmbill-section10007/fy19/FY19-farmbill-faq.pdf.

[71] USDA APHIS Plant Protection Act, Section 7721. Fiscal Year 2019 Spending Plan. https://www.aphis.usda.gov/plant_health/ppa-7721/FY19/fy19-ppdmdpp-spending-plan.pdf.

[72] USDA Forests and Rangelands Healthy Forests Restoration Act of 2003: Summary of Implementation Actions. https://www.forestsandrangelands.gov/resources/overview/hfra-implementation12-2004.shtml.

[73] U.S. Congress Empowering State Forestry to Improve Forest Health Act of 2018. https://www.congress.gov/bill/115th-congress/house-bill/4976.

[74] Aukema J.E., McCullough D.G., Von Holle B., Liebhold A.M., Britton K., Frankel S.J. (2010). Historical accumulation of nonindigenous forest pests in the continental United States. BioScience.

[75] Haack R.A., Britton K.O., Brockerhoff E.G., Cavey J.F., Garrett L.J., Kimberley M., Lowenstein F., Nuding A., Olson L.J., Turner J. (2014). Effectiveness of the international phytosanitary standard ISPM no. 15 on reducing wood borer infestation rates in wood packaging material entering the United States. PLoS ONE.

[76] Leung B., Springborn M.R., Turner J.A., Brockerhoff E.G. (2014). Pathway-level risk analysis: The net present value of an invasive species policy in the US. Front. Ecol. Environ..

[77] Liebhold A.M., Berec L., Brockerhoff E.G., Epanchin-Niell R.S., Hastings A., Herms D.A., Kean J.M., McCullough D.G., Suckling D.M., Tobin P.C. (2016). Eradication of invading insect populations: From concepts to applications. Ann. Rev. Entomol..

[78] Koch F.H., Yemshanov D., Colunga-Garcia M., Magarey R.D., Smith W.D. (2011). Potential establishment of alien-invasive forest insect species in the United States: Where and how many?. Biol. Invas..

[79] Elsensohn J.E., Anderson T., Cryan J.R., Durham T., Gandhi K.J.K., Gordon J., Krell R.K., Pimsler M.L., Rivers A., Spafford H. (2019). From research to policy: Scientists speaking for science. Ann. Entomol. Soc. Am..

[80] Spafford H. (2019). Scientists in the politicoscientific community: Beyond the Lorax. Ann. Entomol. Soc. Am..

[81] Entomological Society of America (ESA) ESA Science Policy Initiatives. https://www.entsoc.org/esa-science-policy.

[82] American Association for the Advancement of Science (AAAS) Science and Technology Policy Fellowships. https://www.aaas.org/programs/science-technology-policy-fellowships.

[83] Bonello P., Campbell F., Cipollini D., Conrad A., Farinas C., Gandhi K.J.K., Hain F., Parry D., Schowalter D., Villari C. Resistance Research and Breeding are Key to Forest Health: Statement of Problem and Request. https://cpb-us-w2.wpmucdn.com/u.osu.edu/dist/7/48782/files/2019/03/TRAG-one-pager-2.0-v67ahs.pdf.

